# Multi-Segment Extendable Soft Manipulator Driven by a Pneumatic–Tendon Coupling Mechanism

**DOI:** 10.3390/biomimetics10100643

**Published:** 2025-09-23

**Authors:** Hongxi Yang, Yufeng Zeng, Zeyu Zhong, Zhiyan Chen, Junxi Zhou, Zhicheng Ling, Ye Chen, Yunquan Li

**Affiliations:** 1 Shien-Ming Wu School of Intelligent Engineering, South China University of Technology, Guangzhou 511442, China; 202264642303@mail.scut.edu.cn (H.Y.); 202264642358@mail.scut.edu.cn (Y.Z.); 202264642389@mail.scut.edu.cn (Z.Z.); 202264642068@mail.scut.edu.cn (Z.C.); 202264642396@mail.scut.edu.cn (J.Z.); 2 School of Microelectronics, South China University of Technology, Guangzhou 511442, China; 202264680107@mail.scut.edu.cn

**Keywords:** continuum robot, soft manipulator, hybrid actuation, kinematic modeling, hybrid control, neural network, trajectory tracking

## Abstract

Continuum robots have garnered significant attention for their high flexibility and adaptability to complex environments. However, achieving the same level of high-precision control as rigid robots remains a significant challenge. This paper introduces an innovative Multi-Segment Extendable Soft Manipulator (MSESM) that employs a pneumatic–tendon hybrid drive mechanism. The design, utilizing off-the-shelf industrial bellows and 3D-printed components, allows the manipulator to achieve an extension ratio of up to 156.85%. By adopting a differential stiffness design, its bending stiffness was increased by approximately 4–5 times, its axial stiffness was increased by approximately 10 times, and its torsional resistance was enhanced, preventing inter-segment coupling during motion. At the control level, this paper proposes a hybrid control method that integrates a Constant Curvature (CC) physical prior with a data-driven neural network. Experimental results show that in tracking rectangular, triangular, and circular trajectories, this hybrid method reduced the average tracking error by 60.43% compared to a purely neural network-based controller, with the error reduction for the rectangular trajectory reaching 74.19%. This research validates a practical and effective approach for creating soft manipulators that successfully merge high flexibility with high-precision control.

## 1. Introduction

As an emerging class of robots, continuum robots have garnered significant attention from both academia and industry in recent years due to their unique compliance and high adaptability to unstructured environments [1,2,3,4]. Unlike traditional rigid-link robots, continuum robots are typically composed of continuously deformable structures that can mimic the movements of biological organisms like elephant trunks [5] and octopus tentacles [6], enabling complex motions such as bending, extension, and torsion [7,8]. This bio-inspired characteristic gives them immense application potential in fields such as medical surgery [1], industrial inspection [9], post-disaster search and rescue [10], and human–robot collaboration [11].

Continuum robots are powered by various actuation methods, with pneumatic [12] and tendon-driven [13] systems being the mainstream technologies, alongside others like shape memory alloys [14] and magnetic actuation [15]. Pneumatic actuation offers advantages such as fast response, high power density, and inherent safety. In contrast, cable-driven actuation allows for remote placement of drive sources, reducing end-effector inertia and providing large output forces. However, the kinematic modeling of continuum robots is highly challenging due to their continuous structures and compliant materials [16]. The piece-wise constant curvature (PCC) model is the most common and simplest modeling approach [17], simplifying the manipulator into a series of arcs with constant curvature. Although the PCC model simplifies analysis, its accuracy is limited, especially in the presence of complex deformations or external loads. Consequently, developing efficient control algorithms is crucial. With advancements in computational power, model-free control strategies [18,19,20,21], which build input–output mappings directly from data, have become a research hotspot. Concurrently, hybrid approaches that combine a baseline physical model with a data-driven residual model to compensate for unmodeled dynamics are gaining traction [22], with recent methods employing advanced techniques like Koopman operator theory [23].

**Figure 1:**
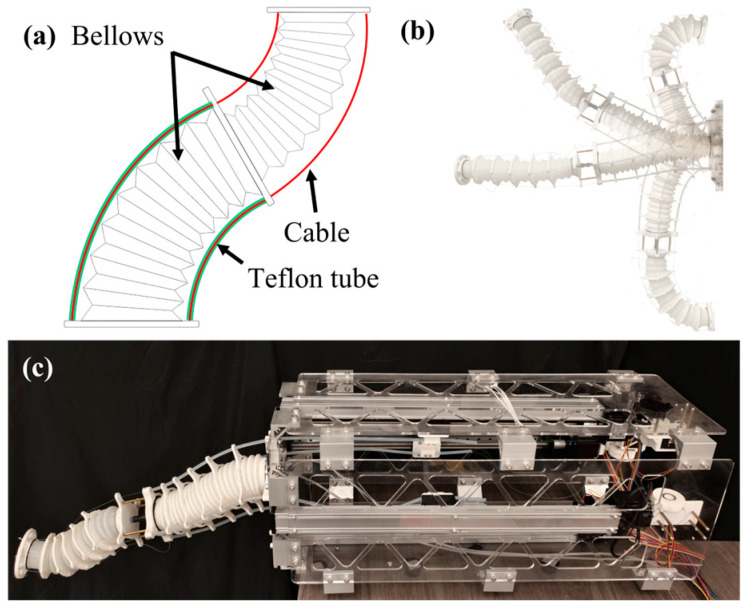
Design concepts of MSESM. (**a**) Tendon drive design (Green wires refer to teflon tubes, red wires refer to cables). (**b**) Deformation of the proposed MSESM prototype. (**c**) Prototype of the MSESM system.

To address the aforementioned challenges, this paper proposes a Multi-Segment Extendable Soft Manipulator (MSESM) driven by a pneumatic–tendon coupling mechanism (Figure 1a). The design allows for adjustable stiffness and length through coordinated control of pneumatic pressure and tendon actuation, providing the manipulator with enhanced flexibility and adaptability in different tasks, as shown by its deformation motion (Figure 1b). The manipulator is based on a bellows structure that integrates off-the-shelf industrial components with 3D printing to simplify the manufacturing process, reduce costs, and enable modular replacement, resulting in the complete robotic system (Figure 1c). To achieve precise motion control, this paper introduces a “prior prediction and data correction” residual learning framework, which combines a structural prior with a data-driven approach. This method is guided by the PCC model, with a neural network compensating for nonlinear deviations, enhancing the model’s accuracy, stability, and generalization in practical tasks. Experimental results show that this hybrid control method reduces the average trajectory tracking error by 60.43% compared to a pure neural network approach and demonstrates extension capabilities, path-following precision, and the ability to adapt to obstacle-crossing tasks.

The main contributions of this paper are as follows:

(1) A novel two-segment soft manipulator is proposed, driven by a pneumatic–tendon hybrid drive mechanism that enables extension, bending, and stiffness modulation. The design integrates off-the-shelf industrial components with 3D printing, reducing manufacturing complexity and cost. The segmented structure with differentiated stiffness effectively prevents motion coupling, ensuring stable control.
(2) A hybrid control method fusing a physical prior with a data-driven approach is constructed. This method uses the PCC model to provide a structural prior that constrains the solution space, while a neural network precisely compensates for model deviations [1]. This strategy effectively avoids common issues in end-to-end learning, such as a lack of interpretability, strong data dependency, and slow convergence, thereby improving control accuracy.

The remainder of this paper is organized as follows. Section 2 introduces the structural design, driving principle, and kinematic modeling of the MSESM. Section 3 presents its extension performance and stiffness characterization. Section 4 details the “prior prediction and data correction” residual learning framework. Section 5 provides multiple experiments to validate the controller’s performance. Section 6 concludes the paper.

## 2. Structural Design and Driving Principle of MSESM

### 2.1. Mechanical Design of the MSESM

**Figure 2:**
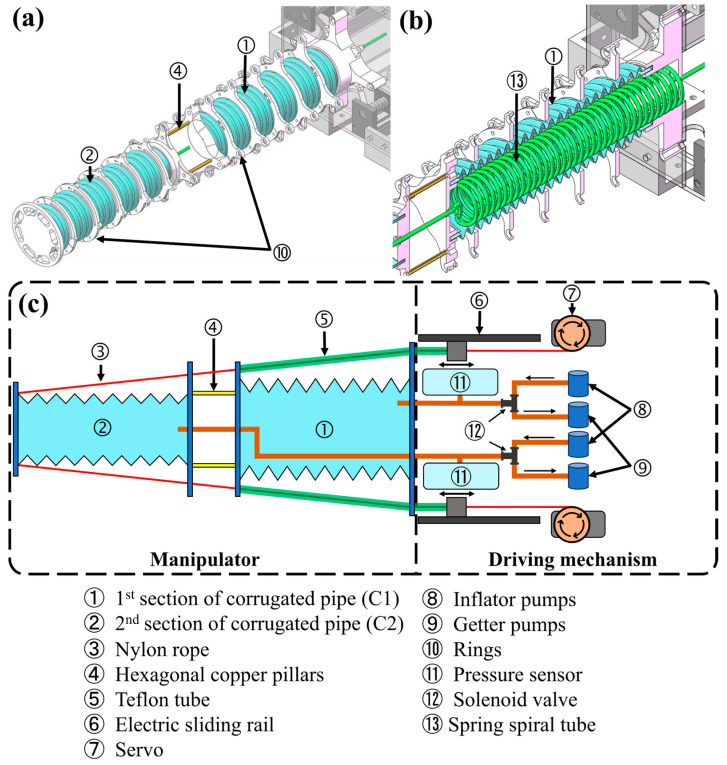
Structural fabrication and design of the proposed MSESM. (**a**) CAD model of MSESM. (**b**) The spiral spring tube hidden within C2. (**c**) Design diagram of MSESM.

The proposed Multi-Segment Extendable Soft Manipulator (MSESM) is structurally divided into two main parts: the actuator part and the driving Mechanism (Figure 2).

Figure 2a,b shows the detailed structure of the MSESM (the 3d model is designed with CAD software SOLIDWORKS 2022). The actuator is the core component that enables the manipulator’s flexible motion. Its main body consists of two silicone bellows (silicone’s properties: hardness: 57 A, tensile strength: 12.55 Mpa, Guangzhou Hongfu Engineering Co., Ltd., Guangzhou, China) of different specifications (C1 and C2 segments) connected in series. To achieve stiffness differentiation and ensure motion decoupling, the C1 segment (near the base) has an inner diameter of 60 mm and a wall thickness of 5 mm, while the C2 segment (at the end) has an inner diameter of 50 mm and a wall thickness of 3 mm, giving the actuator an overall tapered shape. Both segments have an initial length of 30 cm and are connected by hexagonal copper pillars. This coarse-to-fine design not only facilitates the continuous routing of tendons but also ensures that when the C2 segment at the end undergoes large-angle bending, the stiffer C1 segment remains stable, avoiding unnecessary motion coupling.

Each segment of the actuator is uniformly distributed with four driving tendons to control bending motion. Although under the constant curvature model, a three-degree-of-freedom motion (bending angle, bending plane, and extension length) theoretically requires only three tendons, this configuration is a critically constrained system and cannot guarantee positive tendon tension in all poses. Therefore, this design adopts a redundant four-tendon drive scheme. According to the principle n ≥ m + 1 (where m is the number of degrees of freedom and *n* is the number of tendons), for a system with m = 3, at least four tendons are required to ensure the feasibility of the inverse kinematics solution and the robustness of the control. Furthermore, the symmetrical distribution of the four tendons allows the opposing pairs to be kinematically decoupled, effectively reducing control complexity. The positions of the tendons are constrained by 3D-printed PLA fixing rings.

The driving mechanism provides the power for the actuator’s motion. To accommodate the stiffness difference between the two segments, this paper employs different tendon materials and driving methods: The C1 segment uses Teflon tubes (Dongguan Xusheng Plastic and Hardware Products Co., Ltd., Dongguan, China) with an outer diameter of 6 mm and an inner diameter of 4 mm as driving tendons (tensile strength: 20~30 Mpa). Their high axial stiffness provides strong support for the manipulator. These four Teflon tubes are pushed and pulled by four independent linear slides(Dongtai City Ouli Transmission Components Co., Ltd., Dongtai, China) driven by stepper motors, achieving bending control of the C1 segment. The C2 segment uses nylon ropes (Hanting Sports Products Co., Ltd., Weihai, China) with a diameter of 0.8 mm as driving tendons (tensile strength: >70 Mpa). These ropes pass through the Teflon tubes of the C1 segment, effectively reducing friction during motion and ensuring that the movement of the C2 segment does not interfere with the state of the C1 segment. These four nylon ropes are wound on winders driven by servo motors(STS3215, Shenzhen Feite Power Technology Co., Ltd., Shenzhen, China). By controlling the motor’s rotation, the nylon ropes are reeled in and out, thus driving the bending of the C2 segment.

In addition to tendon-driven actuation, the MSESM also integrates pneumatic actuation to achieve axial extension. Each bellows segment is connected to an independent pneumatic circuit, which includes a pump for inflation and a pump for deflation. A sealed chamber equipped with a pressure sensor (BMP280, Xinde Electronics Co., Ltd., Shenzhen, China, accuracy: ±0.12 hPa, sampling rate: 100 Hz) is used to monitor the internal pressure in real-time. A PID control algorithm adjusts the solenoid valve to precisely maintain the pressure at the setpoint, thereby controlling the length of the bellows.

(1) $$\begin{array}{c} u\left(t\right)\;=\;K_{p}\;e\left(t\right)\;+\;K_{i}\;\int_{0}^{t}e\left(\tau \right)\;d\tau \;+\;K_{d}\;\frac{\mathrm{de}\left(t\right)}{\mathrm{dt}}\\ e\left(t\right)=\mathrm{SP}-\mathrm{IP}\\ \mathrm{SP}:\;\mathrm{Set}\;\mathrm{pressure}\\ \mathrm{IP}:\;\mathrm{Instantaneous}\;\mathrm{pressure} \end{array}$$

where *SP* is the set pressure and *IP* is the instantaneous pressure.

By adjusting the state of the solenoid valve, the air chamber is selectively connected to the inflation or deflation pump (Guangfeng Mechanical Equipment Trading Co., Ltd., Kunshan, China). When the measured pressure is below the preset value, the chamber is connected to the inflation pump to increase the pressure, and vice versa. As shown in Figure 2c, to avoid affecting the extension of the C1 segment, the pneumatic tube for C2 is a spiral spring tube (Liu Shi Yiyang Pneumatic Components Trading Co., Ltd., Leqing, China), cleverly hidden inside the cavity of the C1 segment and led out from the base. The bellows and fixing parts are bonded with epoxy resin (Whale World Intelligent Technology Co., Ltd., Changzhou, China) to ensure airtightness.

### 2.2. Forward and Inverse Kinematic Analysis of the MSESM End-Effector (And Avoiding Singularities in Inverse Kinematics)

**Figure 3:**
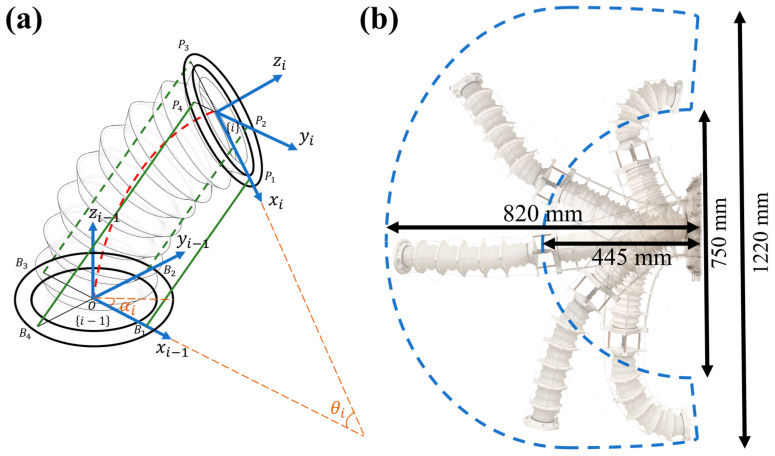
(**a**) Parameters of PCC in one section of the bellow. (**b**) Workspace analysis of MSESM.

For the MSESM, which requires forward kinematic modeling, its structure is a tapered elastic bellows driven by four uniformly distributed tendons at its periphery. It is important to note that although the tapered configuration of the MSESM means its geometry does not fully satisfy the conditions for the constant curvature model, the CC model can still provide effective qualitative analysis for this structure and simplify the subsequent accuracy optimization process. In this model, the constant curvature assumption simplifies the geometric properties of the bellows, making it play an important role in the preliminary evaluation of structural performance, especially when considering driving effects and deformation responses [24]. As shown in Figure 3a, for the i-th bellows segment, there are two different coordinate systems, {i − 1} and {i}, located at the center of the base and the moving platform, respectively.

Specifically, the coordinate system {i − 1} is defined at the base of the bellows, with its $x_{i-1}$ axis pointing to the anchor point of the first driving tendon, and its $z_{i-1}$ axis perpendicular to the base. Correspondingly, the coordinate system {i} is defined at the moving platform of the bellows, with its $x_{i}\;$ axis pointing to the anchor point of the first driving tendon, and its $z_{i}$ axis perpendicular to the moving platform. This coordinate system configuration is used to describe the pose changes of each joint of the bellows during motion, and the kinematic transformation from coordinate system {i − 1} to {i} is represented by a homogeneous transformation matrix. Based on the bending characteristics of the MSESM, two joint variables are introduced to characterize the bending motion of the bellows: the bending angle $\theta_{i}$ and the rotation angle of the bending plane $\alpha_{i}$, where $\theta_{i}\;\in \;[0,\;\pi /2],\;\alpha_{i}\;\in \;[-\pi ,\;\pi ]$. The bending plane OCPB, as shown in Figure 3a, is the bending plane of the bellows and is always perpendicular to the base. We can use this structure to perform quantitative forward and inverse kinematic analysis of the MSESM.

Regarding the forward kinematic analysis of the MSESM, the most critical step is to derive the transformation matrix from the moving platform coordinate system to the base coordinate system using the constant curvature parameters $L_{i}$ (length of the central axis of the bellows), $\theta_{i}$, and $\alpha_{i}$. According to [25], we obtain the formula for the transformation matrix $T_{i-1,i}\left(\alpha_{i},\theta_{i}\right)$ from the moving platform coordinate system {i} to the base coordinate system {i − 1} as:

(2) $$T_{i-1,i}\left(\alpha_{i},\theta_{i}\right)\;=\;e^{{\hat{\xi }}_{i}\theta_{i}}T_{i-1,i}\left(0\right)$$

where ${\hat{\xi }}_{i}\;=\;\left[\begin{array}{cc} {\hat{\omega }}_{i} & v_{i}\\ 0 & 0 \end{array}\right]\;\epsilon \;\mathrm{se}(3)$ represents the twist of the i-th MSESM segment in the coordinate system {i − 1}. Its twist axis is represented as $\xi_{i}\;=\;\left(\;v_{i},{\;\omega }_{i}\right)\;\epsilon \;R^{6\times 1}$, where $v_{i}$ is the translational component of the screw axis, representing its initial position relative to the coordinate system {i − 1}. $\omega_{i}$ is the unit rotation vector, representing the direction of the rotation axis of the i-th bellows segment relative to the coordinate system {i − 1}.

Next, for the terms $T_{i-1,i}\left(0\right)$ and $e^{{\hat{\xi }}_{i}\theta_{i}}$ in the equation, we have:

(3) $$T_{i-1,i}\left(0\right)\;=\;\left[\begin{array}{cccc} 1 & 0 & 0 & 0\\ 0 & 1 & 0 & 0\\ 0 & 0 & 1 & L_{i}\\ 0 & 0 & 0 & 1 \end{array}\right]$$

(4) $$e^{{\hat{\xi }}_{i}\theta_{i}}=\left[\begin{array}{cc} e^{{\hat{\omega }}_{i}\theta_{i}} & \left(I-e^{{\hat{\omega }}_{i}\theta_{i}})(\omega_{i}\times v_{i})+(\omega_{i}\omega_{i}^{T}v_{i}\theta_{i}\right)\\ 0 & 1 \end{array}\right]$$

For the translational component $v_{i}$ and the unit rotation vector $\omega_{i}$ of the screw axis, we have:

(5) $$v_{i}\;=\;L{\left[\begin{array}{ccc} -\frac{1}{2}\cos\alpha_{i} & -\frac{1}{2}\sin\alpha_{i} & \frac{1}{\theta_{i}}-\frac{1}{2}\cot\frac{1}{\theta_{i}} \end{array}\right]}^{T}$$

(6) $$\omega_{i}=\;{\left[\begin{array}{ccc} -\sin\alpha_{i} & \cos\alpha_{i} & 0 \end{array}\right]}^{T}$$

Through the above equations, we can obtain the transformation matrix $T_{i-1,i}\left(\alpha_{i},\theta_{i}\right)$ from the moving platform coordinate system {i} to the base coordinate system {i − 1}. Since the MSESM system contains multiple bellows segments, we need to multiply the transformation matrices of each segment sequentially to obtain the overall forward kinematics model [26]. Specifically, based on the segmented drive structure, the forward kinematics model of the MSESM can be expressed as the product of the transformation matrices of each bellows segment’s kinematics model:

(7) $$T_{0,n}\left(\alpha ,\theta \right)\;=\;T_{0,1}\left(\alpha_{1},\theta_{1}\right)\ldots T_{i-1,i}\left(\alpha_{i},\theta_{i}\right)\ldots T_{n-1,n}\left(\alpha_{n},\theta_{n}\right)$$

Through the transformation matrix, we can obtain the mapping relationship between the multi-segment constant curvature parameters $\left({L_{i},\;\alpha }_{i},{\;\theta }_{i}\right)$ and the end-effector pose variables (x, y, z, ϕ, θ, ψ), which also facilitates the subsequent derivation of the Jacobian matrix. It is worth noting that we use ZYX Euler angles to represent the end-effector’s orientation. Since the workspace of the MSESM does not include pitch angles of $\pm \frac{\pi }{2}$ during the design process, there is no gimbal lock problem. The mapping relationship is as follows:

In three-dimensional space, the forward kinematics model structure can be represented as $T\;=\;\left[\begin{array}{cc} R & p\\ 0 & 1 \end{array}\right]$, where R is a 3 times 3 rotation matrix and p is a 3 × 1 position vector:

(8) $$\begin{array}{c} p\;=\;{\left[\begin{array}{ccc} T_{14} & T_{24} & T_{34} \end{array}\right]}^{T}\\ R\;=\;\left[\begin{array}{ccc} T_{11} & T_{12} & T_{13}\\ T_{21} & T_{22} & T_{23}\\ T_{31} & T_{32} & T_{33} \end{array}\right] \end{array}$$

We can then obtain:

(9) $$\begin{array}{c} x\;=\;T_{14},\;y\;=\;T_{24},\;z\;=\;T_{34}\\ \psi \;=\;\mathrm{atan}2\left(R_{21},R_{11}\right)\\ \theta \;=\;\mathrm{atan}2\left(-R_{31},\sqrt{R_{11}^{2}+R_{21}^{2}}\right)\\ \phi \;=\;\mathrm{atan}2(R_{32},R_{33}) \end{array}$$

Table 1 presents the poses of the end planes obtained by using the aforementioned kinematic modeling methods

Regarding inverse kinematics, for the MSESM structure, its forward kinematics involves the serial transformation of multiple bellows segments, and the motion transformation of each segment depends on multiple joint variables. Although the transformation matrix of each bellows segment can be calculated sequentially through the forward kinematics model, deriving the joint variables directly from the target pose using a global inverse matrix is extremely complex. The forward kinematics of the MSESM includes nonlinear joint variables such as angles and bending planes, and the connection relationship of multiple bellows segments leads to the complexity of the transformation matrix, making it difficult to obtain a global inverse matrix through simple linear transformations. Furthermore, the inverse kinematics problem is often multi-solution, meaning that multiple joint configurations may satisfy the same end-effector pose. Therefore, directly calculating the global inverse matrix not only has the problem of non-uniqueness of the solution but may also fail to obtain a solution due to complex geometric constraints.

To solve these problems in inverse kinematics, we use the pseudo-inverse of the Jacobian matrix instead of the global inverse matrix for local linear estimation. The Jacobian matrix is solved as follows:

For the MSESM structure, its multi-segment joint variables $\left(\alpha_{i},\;\theta_{i},\;L_{i}\right)$ represent the bending angle, bending plane rotation angle, and central axis length of the bellows segment, while the end-effector pose variables are represented by (x, y, z, ϕ, θ, ψ). We use $V_{\mathrm{end}}$ and $\dot{\theta }$ to represent the first derivatives of the pose vector and the joint variable vector, respectively. We can obtain the following equation:

(10) $$V_{\mathrm{end}}\;=\;J(\theta )\dot{\theta }$$

The J(q) in the equation is the Jacobian matrix:

(11) $${J\left(\theta \right)}^{T}\;=\;\left[\begin{array}{cccccc} \frac{\partial x}{\partial \alpha_{i}} & \frac{\partial y}{\partial \alpha_{i}} & \frac{\partial z}{\partial \alpha_{i}} & \frac{\partial \phi }{\partial \alpha_{i}} & \frac{\partial \theta }{\partial \alpha_{i}} & \frac{\partial \psi }{\partial \alpha_{i}}\\ \frac{\partial x}{\partial \theta_{i}} & \frac{\partial y}{\partial \theta_{i}} & \frac{\partial z}{\partial \theta_{i}} & \frac{\partial \phi }{\partial \theta_{i}} & \frac{\partial \theta }{\partial \theta_{i}} & \frac{\partial \psi }{\partial \theta_{i}}\\ \frac{\partial x}{\partial L_{i}} & \frac{\partial y}{\partial L_{i}} & \frac{\partial z}{\partial L_{i}} & \frac{\partial \phi }{\partial L_{i}} & \frac{\partial \theta }{\partial L_{i}} & \frac{\partial \psi }{\partial L_{i}} \end{array}\right]$$

Next, the pseudo-inverse of the Jacobian matrix can be calculated through Singular Value Decomposition (SVD) or the Moore–Penrose pseudo-inverse formula. In general, it can be expressed as:

(12) $$J^{+}\;=\;{\left(J^{T}J\right)}^{-1}J^{T}$$

If the Jacobian matrix is non-square or close to a singular point, the pseudo-inverse can provide the optimal solution in the adjacent state space by minimizing the error.

(13) $$\dot{\theta }=J^{+}\;\cdot \;V_{\mathrm{end}}$$

### 2.3. Mapping and Decoupling Analysis Between Drive Space and Constant Curvature State Space

The MSESM is composed of multiple elastic bellows segments driven by tendons. The motion space of the entire actuator is shown in Figure 3b, with a horizontal extension range from 445 mm to 820 mm, achieving an extension range of 375 mm. The vertical bending range is from 750 mm to 1220 mm. The overall motion space presents a fan-like shape. Given the two independently extendable bellows of the MSESM, its motion flexibility is greatly enhanced compared to other non-extendable continuum manipulators. Since we cannot directly control the change of constant curvature parameters to control the end-effector pose, we must rely on motors to control the tendon lengths to change the constant curvature parameters. Therefore, knowing the mapping relationship between the tendon drive space and the constant curvature parameter state space is necessary for both forward and inverse kinematic analysis of the system.

Each bellows segment can be represented by the joint variables ($\alpha_{i},{\;\theta }_{i},\;L_{i}$), and the length of the driving tendon $l_{\mathrm{ij}}$ can be expressed by these joint variables. Here, i represents the bellows segment number, and j represents the tendon segment number counted from the anchor point pointed to by the $x_{i}$ axis, following the right-hand rule with the $z_{i}$ axis. We can then obtain:

(14) $$l_{\mathrm{ij}}\;=\;2\frac{L_{i}}{\theta_{i}}\sin\frac{\theta_{i}}{2}\;-\;2r\cos\beta_{j}\sin\frac{\theta_{i}}{2}$$

where *r* is the radius of the circle where the anchor points are located, and $\beta_{j}$ is the rotation angle of the tendon relative to the base:

(15) $$\beta_{j}\;=\;\alpha \;+\;\left(j\;-\;1\right)\frac{\pi }{2},\;(j\;=\;1,\;2,\;3,\;4)$$

This formula is derived from the geometric configuration of the four-tendon system, correlating the tendon length with the joint angles that define the bending of each tendon. Since the bellows of the MSESM system are tapered, we use this model as an approximation.

Simultaneously, we can also know that:

(16) $$\begin{array}{c} L_{i}\;=\;\frac{\sum_{j=1}^{4}l_{\mathrm{ij}}}{4}\\ \alpha_{i\;}=\;\mathrm{atan}2\left(\frac{l_{i4\;}-\;l_{i2}}{l_{i3}\;-\;l_{i1}}\right)\\ \theta_{i}\;=\;2\arcsin\left(\frac{\sqrt{{\left(l_{i3}-l_{i1}\right)}^{2}\;+\;{\left(l_{i4}-l_{i2}\right)}^{2}}}{4r}\right) \end{array}$$

In the two-segment MSESM system, the tendon lengths of the two bellows segments influence each other; a change in the length of the first segment’s tendons directly affects the length of the second segment’s tendons. To achieve kinematic decoupling, we need to design a control strategy to ensure that the curvature parameters of each bellows segment are independent, avoiding cross-influence between segments.

Assuming the length of the j-th tendon of the i-th segment is $l_{\mathrm{ij}}$, and the corresponding tendon length of the second segment is $l_{(i+1)j}$, then according to the system’s geometric configuration, when the tendon length $l_{ij}$ changes, the tendon length $\;l_{(i+1)j}$ must be adjusted to ensure the independence of the two segments. The specific adjustment equation is as follows:

(17) $$∆l_{(i+1)j}\;=\;∆l_{\mathrm{ij}}\;\cdot \;k$$

where k is the decoupling scaling factor, used to adjust the mutual influence between the two segments, and is usually obtained through experiments. The motor controller then achieves the required tendon length change by adjusting the encoder values, thereby maintaining the independence of the two tendon segments. The ultimate goal is to make the length of each tendon segment correspond to its desired curvature parameters during the control process.

## 3. Experimental Characterization of the MSESM

### 3.1. Extension Ratio Test

**Figure 4:**
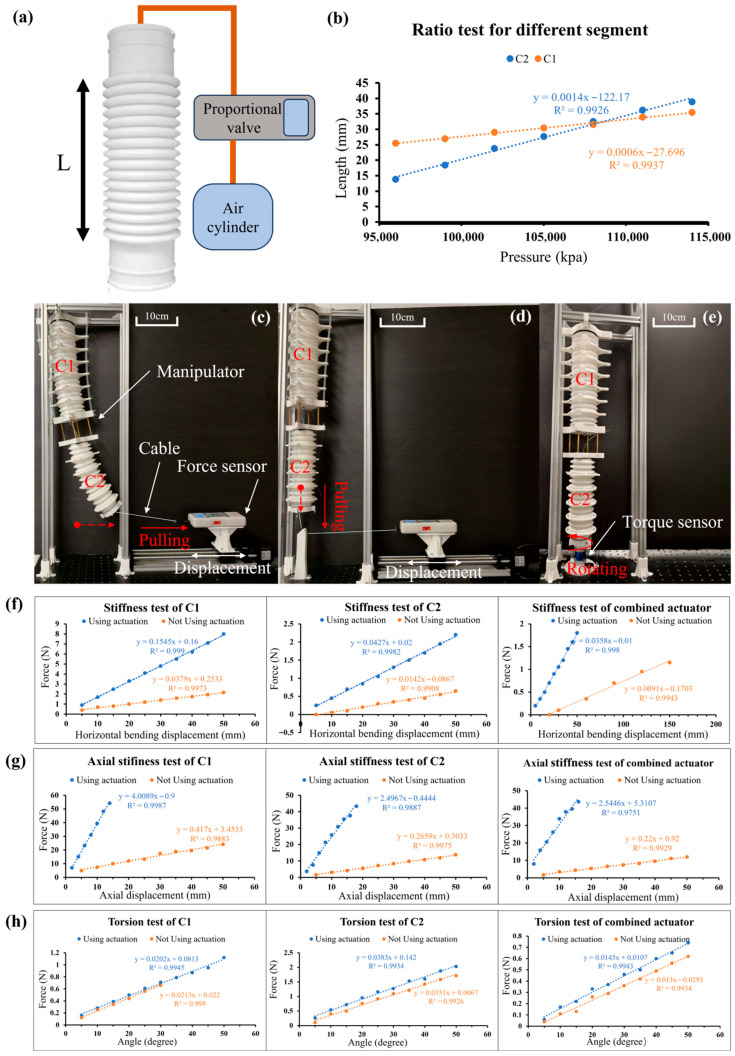
Quantitative characterization of MSESM. (**a**) Ratio test of one bellow. (**b**) Result of the ratio test of each segment. (**c**) Stiffness test of MSESM. (**d**) Axial stiffness test of MSESM. (**e**) Torsion test of MSESM. (**f**) Stiffness test results of different segments under constrained and unconstrained conditions. (**g**) Axial stiffness test results of different segments under constrained and unconstrained conditions. (**h**) Torsion test results of different segments under constrained and unconstrained conditions.

The most significant feature of this actuator is its extension and compression performance. The bellows have a spring-like physical form and can be stretched and compressed. The extension ratios of C1 and C1 are calculated by using the experimental equipment shown in Figure 4a. The length L refers to the length of the corrugated part in the bellows. As shown in Figure 4b, the length changes of the C1 and C2 bellows under different unconstrained pressures were recorded. Under a pressure of 0.96 MPa to 1.2 MPa, the length of C1 ranged from 25.5 cm to 35.5 cm (original length 28.3 cm), and the length of C2 ranged from 13.8 cm to 38.9 cm (original length 24.8 cm). Due to the sealing performance and extension/compression performance of the bellows, the compression ratio of C1 is 90.10% and the extension ratio is 125.44%, while the compression ratio of C2 is 55.65% and the extension ratio is 156.85%. As shown in Figure 4b, the length of the bellows changes linearly with pressure. Since the wall thickness of C2 (3 mm) is thinner than that of C1 (5 mm), the elastic coefficient of C2 is lower than that of C1. Therefore, under the same pressure change, the extension/compression of C2 is less than that of C1.

### 3.2. Stiffness Test of Different Segments

To verify the stiffness difference between the two bellows, the forces under the same displacement were measured and compared, thus validating the decoupling performance between the two bellows. As shown in Figure 4c, the bellows were fixed on a stable aluminum frame, and a cable was connected to a force gauge on the right. The force gauge was mounted on a lead screw slider. Due to the precise movement of the lead screw slider, the force gauge moved 5 mm to the right each time. We tested the stiffness characteristics of C1 and C2 with and without tendon constraints, and the results are shown in Figure 4f. It can be seen that under the same displacement, the force acting on C1 is greater than the force acting on C2, regardless of whether there are constraints. In addition, with the application of tendons, the stiffness of the actuator is enhanced, especially under the constraint of the stiffer Teflon tubes. This phenomenon, where stiffness is actively modulated through a combination of structural constraints and internal pressure, is a key characteristic of hybrid-actuated soft systems and has been explored in other recent works focusing on hyperelastic modeling and pressure-stiffening effects [27].

### 3.3. Axial Stiffness Test

The axial stiffness test was used to examine the effect of the driving tendons on the axial strain of the actuator. The experiment tested the axial stiffness data of C1, C2, and the entire actuator segment separately. As shown in Figure 4d, the bellows are fixed to an aluminum frame with a nylon rope at the end, and the direction of the line is turned 90 degrees to horizontal by an object fixed to the base. The other section of the line is connected to the force gauge, which moved 5 mm to the right each time. Figure 4g shows that when constrained by tendons, the axial stiffness of the actuator is improved. By observing the slope, it can be found that the axial stiffness of the bellows with tendon constraints is about 10 times that of the unconstrained case.

### 3.4. Torsion Test

In the torsion test shown in Figure 4e, the torsional forces of C1, C2, and their combined structure were tested with and without tendon constraints. The test torsion range was from 0° to 50°, with data recorded every 5°. As shown in Figure 4e, a torque sensor was installed at the bottom of the MSESM to measure the torque under different conditions. As can be seen from Figure 4h, after adding tendons, the torsional stiffness of both C1 and C2 improved to varying degrees. Due to the Teflon tube tendons of C2, its torsional resistance was most significantly enhanced. Since C2 is located at the bottom and is first subjected to the torsional force, while C1 is located above and is less affected, the combined data of C1 and C2 is between the two.

## 4. Construction of the “Prior Prediction and Data Correction” Residual Learning Architecture

To improve the accuracy of inverse kinematics solving in high-dimensional nonlinear state spaces, this paper proposes a hybrid modeling method that fuses structural priors with data-driven techniques. This method uses the constant curvature model as a structural guide to construct a “prior prediction and data correction” residual learning framework. While maintaining physical consistency and interpretability, it enhances the model’s accuracy, stability, and generalization ability in practical tasks [28].

In this framework, the end-effector pose vector $y\;\in \;R^{6}$ represents the end-effector pose observed by the vision system (ORBBEC Gemini 2 stereo camera + AR tag), including spatial position (x, y, z) and Euler angle orientation (roll, pitch, yaw). The traditional constant curvature model can provide an approximate mapping from the end-effector state space y to the constant curvature variable space $u\;\in \;R^{n}$ (where *n* is the number of constant curvature variables, 6 in this paper). By solving its inverse kinematics function $f_{\mathrm{cc}}^{-1}\left(y\right)$ using the pseudo-inverse of the Jacobian matrix at a fixed reference point, an initial predicted solution is obtained:

(18) $$u^{\prime }\;=\;f_{\mathrm{cc}}^{-1}\left(y\right)$$

Since the CC model only considers ideal circular arc deformation and ignores real physical factors such as material nonlinearity, tendon friction, uneven tension, and inter-segment coupling, and because this solution is a local linearized estimation result, its output u′ has a systematic deviation from the actually collected drive quantity u. The residual e is defined as:

(19) $$e\;=\;u-u^{\prime }$$

To correct this deviation, this paper introduces a neural network module **f**(·) to learn the nonlinear mapping from the end-effector state y to the residual e, i.e., to construct the residual function:

(20) $$f(y)\;\approx \;u-f_{\mathrm{cc}}^{-1}\left(y\right)$$

The network training objective is to minimize the mean squared error between the predicted residual and the actual residual, thereby obtaining a compensation model. The final drive prediction result can be written as:

(21) u^  = fcc−1(y) + f(y)

The above model structure has two advantages: on the one hand, $f_{\mathrm{cc}}^{-1}\left(y\right)\;$ provides an interpretable and structurally clear initial prediction, effectively reducing the learning difficulty for the neural network; on the other hand, the neural network only needs to fit the nonlinear residual caused by non-idealities, which greatly reduces the model complexity and improves data efficiency, convergence speed, and stability.

In the early stage of training, to improve the initial accuracy of the prior prediction, this paper adopts a linearized estimation strategy based on the pseudo-inverse of the Jacobian matrix. Let the target end-effector pose be y_target and the fixed reference pose be y_fixed. Through local first-order linear approximation, the drive increment Δu is obtained:

(22) $$\Delta u\;=\;J^{+}(y\_\mathrm{fixed})\;\cdot \;(y\_\mathrm{target}-y\_\mathrm{fixed})$$

where $J^{+}(y\_\mathrm{fixed})$ is the Moore–Penrose pseudo-inverse of the Jacobian matrix corresponding to the constant curvature model at the fixed reference end-effector state. This linear prediction result is used to initialize $f_{\mathrm{cc}}^{-1}\left(y\right)$ and provide an initial reference for the network’s residual, further narrowing the training space and improving the convergence rate of the residual network.

The core of the entire residual learning framework lies in using a physical model to provide a structural prior, achieving contraction and regularization of the target solution space, and then having a neural network compensate for non-ideal deviations to achieve precise modeling. This structure effectively avoids problems that may be introduced by end-to-end black-box learning, such as a lack of interpretability, strong data dependency, and slow training convergence. It is a “white-box + black-box” hybrid modeling strategy that combines physical rationality with data adaptability

### 4.1. Reasons for Choosing the Constant Curvature Model

To enhance modeling accuracy and control robustness with limited sample data, we propose to integrate the constant curvature (CC) model with a neural network (NN) to construct a data-driven hybrid modeling framework guided by physical priors. This method uses the initial structural mapping capability provided by the CC model as a guide, injecting physically consistent prior knowledge into the neural network. This improves model accuracy and enhances the stability and generalization ability of the inverse kinematics solution. The specific implementation will be detailed in subsequent subsections.

Although a single CC model, with its basic assumption that each segment of the manipulator can be approximated as a constant curvature circular arc, can provide a good description under ideal conditions, its accuracy is often limited in real-world environments due to complex physical effects such as gravity, self-weight, external loads, and friction coupling. Especially in tendon-driven or pneumatically driven operations, the CC model cannot fully capture behaviors like tendon tension distribution and nonlinear deformation of elastomers. Furthermore, because its Jacobian matrix may experience a rank drop in certain configurations (such as fully extended or highly symmetric configurations), it leads to Jacobian singularities in the inverse kinematics solution, manifested as limited end-effector degrees of freedom, non-unique solutions, or diverging joint velocities [29].

However, choosing the CC model as the structural prior in hybrid modeling still has multiple theoretical and engineering advantages. First, compared to variable curvature (VC) models, Cosserat rod theory, or lumped-parameter models [30], the CC model has a clearer input–output mapping relationship, and its structural parameters (such as curvature, module length, and direction angle) can be related to drive variables like tendon length or chamber pressure through concise analytical expressions. This mapping is highly physically interpretable and computationally inexpensive. By constructing the pseudo-inverse of the Jacobian matrix, a local linearized prediction can be generated and embedded as a guiding term in the neural network training, forming a “prior prediction + data correction” residual learning architecture. Second, the CC model has the ability to screen for reasonable boundaries of the drive space, effectively excluding invalid input combinations during sampling, preventing harmful outputs (such as simultaneous excessive contraction of diagonal tendons), avoiding the risk of structural damage, and improving sampling efficiency.

More critically, although the CC model has certain deviations in modeling accuracy, it can fairly accurately capture the dominant deformation trend of the continuum manipulator, providing structural guidance for the neural network’s residual learning. Leveraging this feature, the fused model can further improve modeling accuracy and numerical robustness based on a large sample size (such as the approximately 9000 training samples in this study). Specifically, the approximate solution provided by the physical prior can serve as an initial approximation for the neural network, allowing the network to focus mainly on fitting the nonlinear residual part, thereby reducing the learning burden, lowering the risk of overfitting, and accelerating convergence. In addition, near singular configurations where the Jacobian matrix degenerates, this hybrid structure also exhibits stronger stability and generalization ability, enhancing the model’s adaptability and fine control capability in complex state spaces.

Therefore, the CC model was chosen in this paper as the physical guidance module for hybrid modeling, representing an optimal trade-off between model interpretability, numerical efficiency, and control-friendliness.

### 4.2. Sampling and Neural Network Model Construction

**Figure 5:**
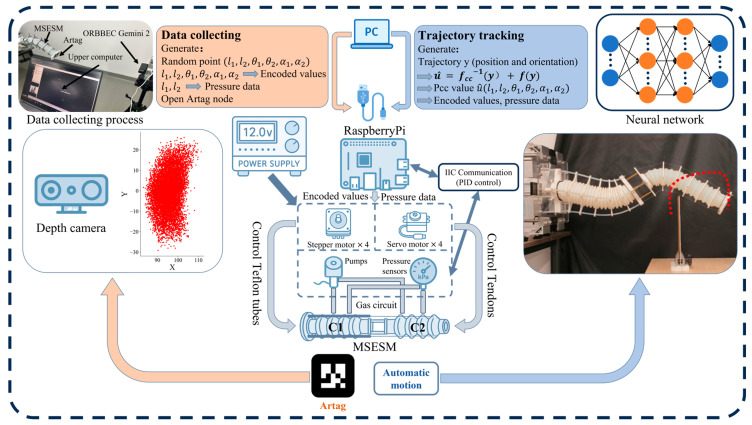
The system structure of the data collection process and trajectory tracking experiment.

To implement the fusion modeling strategy of the constant curvature model and the neural network, this paper constructed a sample set containing 9464 sets of end-effector pose data, with each sample corresponding to the spatial configuration of a two-segment constant curvature structure. Each segment’s configuration is uniquely determined by three parameters: bending angle theta, bending plane direction alpha, and segment length l. The length of the first segment, l_1, varies from 25.5 cm to 28.3 cm, and the length of the second segment, l_2, varies from 13.8 cm to 24.8 cm, covering the main extension range of the system in actual operation. The bending angle theta is uniformly sampled from 0° to 30°, and the bending plane direction alpha ranges from −180° to 180°, achieving full omnidirectional configuration coverage in three-dimensional space. The system structure for this process is shown in Figure 5. Simultaneously, we convert the parameters from the constant curvature space to the drive encoder values of the actuator through the mapping relationship between the drive space and the constant curvature state space, thus achieving quantitative control.

The reason for limiting the bending angle theta to the range of 0° to 30° is based on the following three considerations: First, when theta exceeds 30°, the nonlinear deformation, material stress distribution, and local structural instability experienced by the system increase, leading to a sharp decline in the fitting accuracy of the constant curvature model. Second, within this angular range, the system’s Jacobian matrix maintains good rank stability, avoiding singularity issues, which is conducive to the numerical convergence and stability of the pseudo-inverse solution in inverse kinematics. Third, the end-effector spatial poses required for actual tasks are mostly concentrated within this angular range, which has good representativeness and task coverage capability.

During the data collection process, the target configuration is generated by controlling the tension combination of the four tendons. The real pose of the system’s end-effector is obtained using an ORBBEC Gemini 2 stereo camera(Shenzhen Yabo Intelligent Technology Co., Ltd., Shenzhen, China) in conjunction with the AR Tag localization function under the ROS platform, including the position vector (x, y, and z) and orientation angles (roll, pitch, and yaw). Each sample point is uniformly distributed in the six-dimensional parameter space (θ_1_, α_1_, l_1_, θ_2_, α_2_, and l_2_), and the sample set has good spatial coverage, configuration diversity, and numerical stability.

This dataset not only provides sufficient support for building a forward model from constant curvature parameters to end-effector pose. The next section will experimentally validate the positioning accuracy, robustness, and network convergence speed of this residual learning architecture based on the collected 9464 sets of constant curvature space samples, and compare it with traditional models.

Based on the sampling, we constructed a Feedforward Neural Network (NN) to map from the end-effector pose to the constant curvature model parameters. The network input is the end-effector’s position (x, y, and z) and Euler angles (θ, φ, and ψ), and the output is the incremental parameters of the constant curvature model ($\Delta l_{1},\;\Delta l_{2},\;\Delta \theta_{1},\;\Delta \theta_{2},\;\Delta \alpha_{1},\;\mathrm{and}\;\Delta \alpha_{2}$). The network contains four hidden layers with 256, 512, 512, and 256 nodes, respectively, and uses the ReLU activation function. The network is trained through supervised learning using a dataset gathered via the process illustrated on the left side of Figure 5. During data collection, a host computer randomly generates the six parameters of the constant curvature model for both segments. These parameters are then converted into corresponding tendon lengths using the model’s inverse matrix, while the target air pressure is calculated based on a linear function of the desired actuator length. These low-level commands are then sent to the Raspberry Pi. The Raspberry Pi controls the motors, moving the end-effector to the target pose, while a stereo camera records the resulting spatial coordinates and orientation. This recorded data, pairing actuator commands with ground-truth poses, forms the training set.

Once trained, the network serves as the core of the trajectory tracking control loop, shown on the right side of Figure 5. This loop is managed by the integrated mechatronics system, with a Raspberry Pi 4B acting as the central controller. The network’s output parameters are processed by the Raspberry Pi (Yue Dong Trading Co., Ltd., Shenzhen, China), which interfaces with all hardware components to translate high-level commands into physical motion. Communication with the pressure sensors is handled via the I2C protocol, allowing for real-time monitoring of the pneumatic system. The Raspberry Pi then uses its GPIO pins to manage the various actuators: it controls eight motor drivers (four for the stepper motors and four for the servo motors) to manipulate the tendons, and it operates a bank of solenoid valves via relay modules to precisely regulate airflow from the pumps. This integrated mechatronic design ensures that the abstract control signals from the neural network are efficiently and accurately converted into the coordinated pneumatic and tendon-driven actions of the manipulator.

## 5. Performance Demonstration of the MSESM

After applying the control method proposed in this paper to the MSESM system, it is possible to achieve efficient and precise regulation of its multi-degree-of-freedom, multi-segment flexible structure. Figure 5 shows its control strategy. Based on a predetermined trajectory input, the system first calculates the target pose parameters for each segment of the soft manipulator through the neural network. Subsequently, the control system converts the output parameters into control commands for air pump pressure and motor position. The air pump uses PID control to achieve precise regulation of air pressure, driving the soft actuator to bend; the motors control the extension or contraction of the manipulator through the tendon-driven method. Finally, the manipulator completes the motion along the desired trajectory, achieving flexible and efficient trajectory tracking control.

To further verify the control performance of this method and the system’s environmental adaptability, the following section will demonstrate its performance and capabilities in trajectory tracking and obstacle-crossing tasks.

### 5.1. Multimodal Motion Performance Demonstration

**Figure 6:**
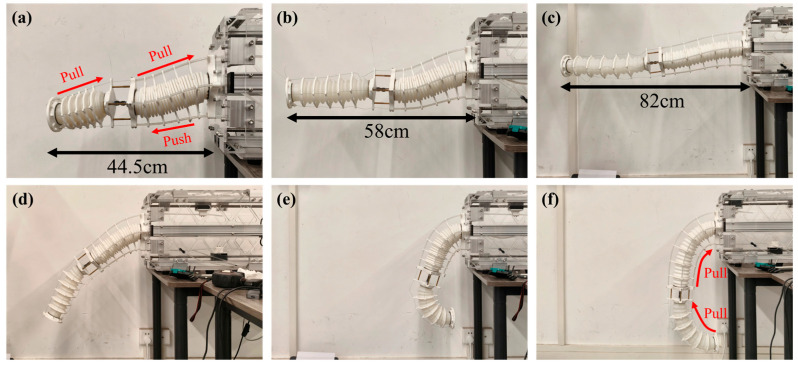
Multimodal motion performance demonstration. (**a**) Full contraction state. (**b**) Original state. (**c**) Full expansion state. (**d**) Half-bent state. (**e**,**f**) Completely bent state.

To visually demonstrate the flexibility and multi-degree-of-freedom motion capability of the proposed Multi-Segment Extendable Soft Manipulator (MSESM), this section conducts a series of key morphology demonstration experiments. The MSESM adopts an innovative pneumatic–tendon hybrid drive structure. By coordinately controlling the air pressure to adjust the arm length and controlling the tendons to adjust the posture, it achieves complex spatial motion capabilities, greatly expanding its workspace and environmental adaptability. Figure 6 shows six typical morphologies of the manipulator under different drive states.

(a) Full Contraction State: This morphology demonstrates the MSESM’s maximum axial compression capability. By evacuating the internal air pressure of the C1 and C2 bellows to below atmospheric pressure (0.9 MPa and 0.96 MPa, respectively) and uniformly tightening all tendons, the manipulator reaches its shortest length. In this state, the manipulator remains straight along its central axis, showcasing its ability to contract in confined spaces.

(b) and (c) Original and Extended States: These two figures demonstrate the manipulator’s length control capability in the horizontal direction against gravity. To maintain a horizontal posture, the upper tendons always apply a certain tension to balance the manipulator’s self-weight. In Figure 6b, the internal and external air pressures of the bellows are basically balanced (about 1.01 MPa), and the manipulator is at its natural length. In Figure 6c, by pressurizing the chamber (C1: 1.2 MPa, C2: 1.1 MPa), the arm length is increased. This clearly shows that by decoupling the control of air pressure and tendons, the MSESM can achieve a large range of extension while maintaining its posture, reflecting the flexibility of its design.

(d) Downward Bending 45° State: This morphology is one of the manipulator’s more natural hanging postures under gravity. In this state, the overall force on the tendons is relatively small. Since the C1 segment’s tendons are made of Teflon tubes with higher stiffness, when the manipulator bends downwards, the lower Teflon tubes are subjected to compressive force, while the upper tendons bear tensile force, working together to stably maintain a 45° bending angle.

(e) and (f) The 180° Fully Bent State: These two morphologies demonstrate the manipulator’s extreme bending capability, with its end plane being 180° relative to its base. This requires applying great tension to the lower tendons of the C1 and C2 segments. The Teflon tubes of the C1 segment, due to their rigid characteristics, provide strong support to the base, making the entire bending arc smooth and controllable. The only difference between states (e) and (f) is the arm length. The internal pressure in Figure 6e (C1: 1.1 MPa, C2: 1.05 MPa) is higher than in Figure 6f (C1: 1.0 MPa, C2: 0.95 MPa), proving that the manipulator can independently adjust its length even in an extreme bending posture.

This series of experiments fully validates the effectiveness of the MSESM design. Its pneumatic–tendon hybrid drive mechanism endows the system with decoupled control of extension and bending, while the segmented structural design and coordinated control strategy enable it to generate complex spatial configurations, laying a solid foundation for subsequent high-level tasks such as high-precision trajectory tracking and flexible obstacle crossing [31].

### 5.2. Trajectory Tracking Experiment

**Figure 7:**
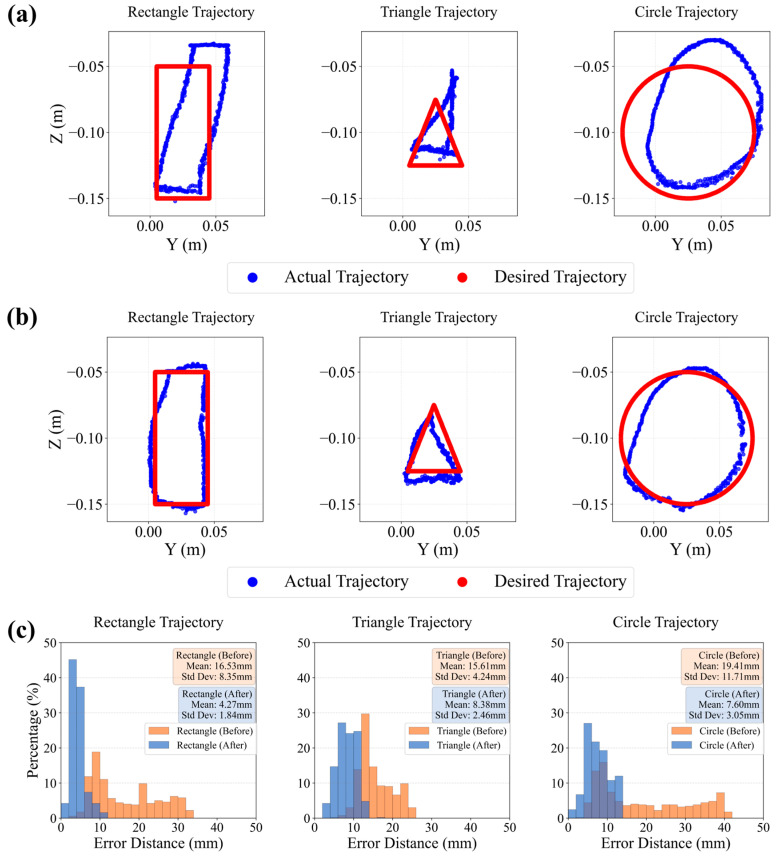
The results of different trajectory dynamic tracking experiments. (**a**) Unoptimized version of actual and target trajectory tracking comparison of different graphics (rectangular, triangle, and circle). (**b**) Optimized version of actual and target trajectory tracking comparison of different graphics (rectangular, triangle, and circle). (**c**) Comparison of the mean errors and standard deviations of the optimized and unoptimized versions of different graphics.

The results of different trajectory dynamic tracking experiments are shown in Figure 7. (a) Unoptimized version of actual and target trajectory tracking comparison of different graphics (rectangular, triangle, and circle). (b) Optimized version of actual and target trajectory tracking comparison of different graphics (rectangular, triangle, and circle). (c) Comparison of the mean and maximum errors of the optimized and unoptimized versions of different graphics. These results are also shown in the second part of the Supplementary Video.

This experiment aims to verify the control accuracy and robustness of the proposed “constant curvature model guidance + neural network residual learning” hybrid modeling method in the trajectory tracking task of a continuum manipulator. We designed three typical two-dimensional target trajectories: a rectangle (100 mm long, 40 mm wide), an isosceles triangle (40 mm base, 50 mm height), and a circle (50 mm radius). Each trajectory consists of 500 equally spaced target points, with a 2 s execution time interval between adjacent points.

The experiment compared the performance of two control strategies: one using only a neural network to directly predict the drive quantities from the end-effector pose (the traditional model), and the other being the method proposed in this paper, which first calculates an initial solution through the constant curvature model and then uses a neural network to learn the residual for fine-tuning. The experiment used a stereo camera and AR tags for real-time tracking of the end-effector pose and recorded the error between the actual and desired trajectories.

The results, as depicted in Figure 7 and the accompanying tables, provide a comprehensive validation of the proposed hybrid method’s superiority. Visually, the trajectory plots show that the traditional model exhibited significant deviation, while the hybrid method produced much smoother trajectories that more closely followed the target paths. This observation is substantiated by a detailed quantitative analysis. The hybrid method significantly reduced the mean tracking error from 16.53 mm to 4.27 mm for the rectangular path, from 15.61 mm to 8.38 mm for the triangular path, and from 19.41 mm to 7.60 mm for the circular path. Furthermore, the error’s variability was also substantially reduced (see Table 2), with the standard deviation decreasing from 8.35 mm to 1.84 mm for the rectangle, from 4.24 mm to 2.46 mm for the triangle, and from 11.71 mm to 3.05 mm for the circle. A t-test confirmed these improvements are statistically significant, yielding *p*-values of 2.39 × 10^−127^, 1.37 × 10^−150^, and 7.87 × 10^−77^ for the rectangular, triangular, and circular trajectories, respectively.

To assess the system’s consistency, repeatability tests were conducted over multiple trials, with the results detailed in Table 3. The low average Dynamic Time Warping (DTW) distances confirm that the manipulator’s movements are highly repeatable. For the traditional (unoptimized) controller, the average DTW distances were 0.0433 m for the rectangle, 0.0355 m for the triangle, and 0.0698 m for the circle. The hybrid controller demonstrated similarly high consistency, with DTW distances of 0.0433 m, 0.0361 m, and 0.0360 m for the respective shapes.

Based on the comprehensive experimental results, this section provides robust validation for the proposed hybrid control strategy. The key finding is that integrating a constant curvature (CC) physical prior with a neural network for residual correction yields a demonstrably more accurate and reliable performance than a purely data-driven approach. This was evidenced by a significant reduction in both the mean tracking error and its variability across all tested trajectories, an improvement that was confirmed to be statistically significant through *t*-tests. Furthermore, repeatability trials demonstrated the high consistency of the manipulator’s movements. Therefore, this section concludes that the hybrid framework is a superior and more robust method for controlling the complex, multi-degree-of-freedom movements of the MSESM.

### 5.3. Three-Dimensional Helical Trajectory Tracking Experiment

To further evaluate the performance of the control system in a more complex spatial manipulation task, a three-dimensional helical trajectory tracking experiment was conducted. This task requires coordinated, simultaneous control over all the manipulator’s degrees of freedom and serves as a more rigorous test of the controller’s capabilities compared to 2D planar tasks. The same two control strategies were compared: the traditional, pure neural network (NN) model and the proposed hybrid constant curvature (CC) and NN model.

**Figure 8:**
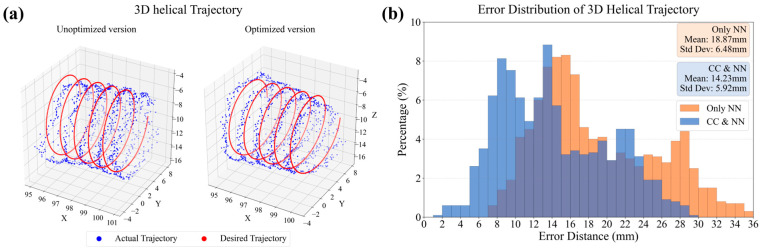
The results of the 3D helical trajectory tracking experiment. (**a**) Unoptimized version and optimized version of the actual and desired trajectory tracking comparison. (**b**) Comparison of the mean and maximum errors of the optimized and unoptimized versions.

The results of this experiment are presented in Figure 8. Figure 8a provides a 3D visualization of the tracking performance, comparing the desired helical path to the actual trajectories produced by both control methods. A quantitative comparison of the average tracking errors is shown in the bar chart in Figure 8b.

This experiment indicates that the hybrid CC and NN model maintained its superior tracking accuracy even in this more demanding 3D task. The pure NN controller resulted in an average tracking error of 1.6593 cm. In contrast, our proposed hybrid model achieved an average tracking error of 1.4231 cm, representing a performance improvement of 14.2%. In addition to improving the average accuracy, the hybrid model also reduced the variability of the tracking error, with the standard deviation decreasing from 6.48 mm to 5.92 mm. This demonstrates the robustness of the hybrid control framework and its effectiveness in guiding the manipulator through complex paths in three-dimensional space, further validating the benefits of incorporating a physical prior into the control architecture.

### 5.4. Obstacle Crossing Test

To further validate the spatial adaptability and coordinated control capability of the proposed MSESM system’s multi-segment flexible structure in complex environments, this section designed and implemented a typical obstacle-crossing experiment. This experiment aims to simulate a flexible exploration task in an unstructured scene. By setting an obstacle, it examines how the system can autonomously complete a series of continuous actions, such as compression, lifting, crossing, extension, and landing, without relying on a rigid end-effector structure, embodying the unique morphological reconfiguration and path adaptation capabilities of continuum robots.

The experiment used the two-segment, multi-tendon driven MSESM designed in this paper, which possesses high compressibility and extendibility. The experimental scene is shown in Figure 8, with a vertical baffle (about 25 cm high) placed 40 cm from the robot’s base to simulate a local obstacle in a real environment. To move from the right side of the baffle to the left, the MSESM’s end-effector must undergo a series of coordinated actions: first, compress to reduce its total length to avoid touching the obstacle, then lift to pass over the top of the obstacle, followed by extending forward, and finally landing to complete the task objective. This result is demonstrated in the third part of the Supplementary Video.

In terms of control strategy, this experiment employed the “constant curvature model + neural network residual learning” hybrid modeling method proposed in this paper. By setting a series of intermediate target points in the host computer (such as compression point, lifting point, crossing point, and landing point), the control system automatically calculates the target length for each of the eight tendons at every moment. For path generation, the neural network is responsible for predicting the residual between the constant curvature parameters and the end-effector pose. By correcting the prior model’s error, it achieves a high-precision mapping from the target trajectory to the tendon drive space.

**Figure 9:**
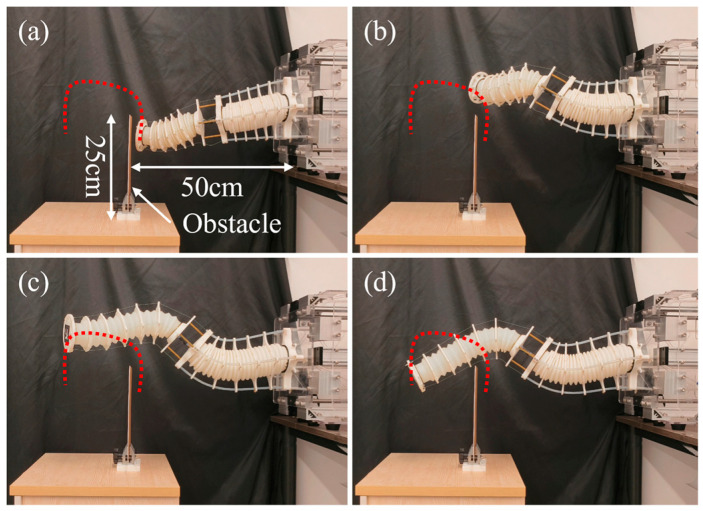
Different stages when MSESM is getting over the obstacle (Dashed line represents the trajectory). (**a**) MSESM is about to encounter an obstacle when in a contracted state. (**b**) The MSESM end rises up and reaches the top of the obstacle. (**c**) The end of the MSESM continues to move to the left while maintaining a high position. (**d**) The end of the MSESM curves downward and reaches the other side of the obstacle.

The action sequence (1) to (4) in Figure 9 shows the state evolution of the MSESM actuator throughout the obstacle-crossing process. The actuator first enters a compressed state (Figure 9a), then gradually stretches the C1 segment and lifts the C2 segment (Figure 9b), continues to extend the C2 segment after successfully crossing the obstacle (Figure 9c), and finally lowers the end-effector platform to complete the path objective (Figure 9d). Throughout the process, the system maintained the decoupling of inter-segment motion, avoiding interference from the front segment due to force transmission, and achieved continuity and stability of the spatial trajectory.

Compared to traditional control methods, this experiment highlights the advantages of the modeling strategy proposed in this paper in solving multi-segment coupled control problems. Even in a strongly constrained environment with obstacles, the control system can still solve complex input–output relationships in real-time and ensure the physical feasibility of the motion path and the reasonableness of the tendon tension distribution. More importantly, this experiment demonstrates the MSESM system’s path self-adaptation and configuration reconfiguration capabilities in a typical “explore-cross-return” task, providing a reliable basis for the practical application of continuum robots in scenarios such as search and rescue and medical intervention.

## 6. Conclusions

This paper successfully designed and implemented an innovative Multi-Segment Extendable Soft Manipulator (MSESM), whose core lies in its pneumatic–tendon hybrid drive structure and segmented differential stiffness design. Through this design, the manipulator’s bending stiffness was increased by approximately 4–5 times and its axial stiffness by approximately 10 times, while its torsional resistance was also enhanced, effectively preventing motion coupling. The manipulator demonstrated a large workspace, with a horizontal extension range of 445 mm to 820 mm and a vertical bending range of 750 mm to 1220 mm. On the control level, our proposed hybrid control framework, which fuses a constant curvature (CC) physical prior with data-driven residual learning, showed superior performance. In trajectory tracking tasks, it reduced the average error by 60.43% compared to a pure neural network method, with the error reduction for the rectangular trajectory reaching as high as 74.19%. Furthermore, in the obstacle-crossing test, the MSESM successfully completed a series of coherent, complex actions, demonstrating its significant application potential in unstructured environments.

Despite these positive results, this study has several limitations. First, although the differential stiffness design provides some support, the inherent compliance of the MSESM makes it susceptible to gravity-induced droop in extended horizontal postures. Additionally, the maximum bending angle of the current prototype is constrained, which limits the overall workspace. Second, while the hybrid control strategy significantly improved accuracy, there is still room for improvement, and the model’s generalization ability to tasks and configurations far outside the training distribution remains a challenge. Part of the residual error stems from factors not fully compensated for by the model, such as friction between the tendons and their conduits and the plastic deformation of the tendons, which are difficult to eliminate entirely with open-loop predictive control. Finally, the current control framework relies on pose feedback from an external vision system for open-loop prediction rather than real-time, closed-loop iterative control based on proprioceptive sensors. This limits the system’s ability to respond quickly to dynamic disturbances. A detailed comparison of the MSESM’s performance against other similar continuum manipulators can be found in Table S2 [4,7,32,33,34,35] and Table S3 [7,32] in the Supplementary Materials.

Future work will be directed at addressing these limitations. First, we will explore combining more precise kinematic models (such as variable curvature models or Cosserat rod theory) as the physical prior with neural networks to systematically compare the performance impact of different base models on the hybrid control framework. Second, we plan to integrate more sensors onto the manipulator’s body (e.g., flexible angle sensors, distributed pressure sensors) to obtain real-time proprioceptive feedback, enabling true closed-loop iterative control and improving the system’s robustness. Future research will also include a more in-depth analysis of the system’s nonlinear characteristics, potentially using tools like Poincaré mapping to better understand its dynamic behavior under various conditions. Finally, optimizing the control algorithm will be a key focus, which includes not only exploring model predictive control or reinforcement learning but also conducting systematic hyperparameter optimization to improve the model’s training efficiency and convergence speed, as well as making material or structural improvements to increase inherent stiffness and mitigate the adverse effects of gravity.

## Figures and Tables

**Table 1:** Samples using forward kinematics analysis.

Cases	Driving Cable Lengths (mm)	Joint Variables ($\alpha_{i},\theta_{i},L_{i}$)	Poses of the End Platform
1	300, 300, 300, 300	0, 0, 0	$\left[\begin{array}{cccc} 1 & 0 & 0 & 0\\ 0 & 1 & 0 & 0\\ 0 & 0 & 1 & 550\\ 0 & 0 & 0 & 1 \end{array}\right]$
	250, 250, 250, 250	0, 0, 0	
2	266, 276, 294, 284	0.3, 0.5, 280	$\left[\begin{array}{cccc} 0.9023 & 0.0623 & 0.6272 & 174.3803\\ 0.0022 & 0.8840 & 0.0765 & 42.1458\\ -0.6162 & -0.0681 & 0.7872 & 435.5178\\ 0 & 0 & 0 & 1 \end{array}\right]$
	196, 202, 205, 198	−0.4, 0.2, 200	
3	335, 335, 305, 305	0.8, −0.7, 320	$\left[\begin{array}{cccc} 1.1403 & 0.4270 & 0.4540 & -74.3875\\ 0.6177 & 0.7814 & 0.1945 & -103.4366\\ -0.0740 & 0.0768 & 0.9311 & 461.2715\\ 0 & 0 & 0 & 1 \end{array}\right]$
	158, 168, 202, 192	0.5, 1.0, 180	

**Table 2:** Reduction of errors and statistical significance tests.

	NN (m)	CC Combined with NN (m)	Reduction of Errors	*p*-Value
Rectangular	0.0165	0.0043	74.19%	2.39 × 10^−127^
Triangle	0.0156	0.0084	46.27%	1.37 × 10^−150^
Circle	0.0194	0.0076	60.84%	7.87 × 10^−77^

**Table 3:** Repeatability results across multiple trials.

	Centroid Deviation (m)	Maximum Centroid Deviation (m)	DTW (m)
Rectangular (Unoptimized)	0.0020	0.0162	0.0433
Triangle (Unoptimized)	0.0018	0.0087	0.0355
Circle (Unoptimized)	0.0056	0.0395	0.0698
Rectangular (Unoptimized)	0.0026	0.0091	0.0433
Triangle (Unoptimized)	0.0018	0.0072	0.0361
Circle (Unoptimized)	0.0017	0.0080	0.0360

## Data Availability

The data presented in this study are available in the main text and Supplementary Materials.

## Supplementary Materials

The following supporting information can be downloaded at: https://www.mdpi.com/article/10.3390/biomimetics10100643/s1, Figure S1: CAD model of MSESM; Table S1: Parts list of MSESM; Table S2: The accuracy and workspace comparison of different continuum robots; Table S3: The stiffness performance and rate of change comparison of different continuum robots; Video S1: Supplementary Video.

## Abbreviations

The following abbreviations are used in this manuscript:

MSESM	Multi-Segment Extendable Soft Manipulator
