# Supplementary material for: Multi-Segment Extendable Soft Manipulator Driven by a Pneumatic–Tendon Coupling Mechanism
Source: Biomimetics (Basel). 2025 Sep 23;10(10):643. doi: 10.3390/biomimetics10100643 (PMC12561715; doi:10.3390/biomimetics10100643)
Supplement: Supplementary file 1 [file biomimetics-10-00643-s001.zip › Supplementary materials.pdf]

Supporting Information

Multi-Segment Extendable Soft Manipulator Driven by a Pneumatic–Tendon Coupling Mechanism

Hongxi Yang [1,†], Yufeng Zeng [1,†], Zeyu Zhong [1,‡], Zhiyan Chen [1,‡], Junxi Zhou [1,‡], Zhicheng Ling [2,‡], Ye Chen [1,*] and Yunquan Li [1,*]

[1] Shien-Ming Wu School of Intelligent Engineering, South China University of Technology, Guangzhou 511442, China; 202264642303@mail.scut.edu.cn (H.Y.); 202264642358@mail.scut.edu.cn (Y.Z.); 202264642389@mail.scut.edu.cn (Z.Z.); 202264642068@mail.scut.edu.cn (Z.C.); 202264642396@mail.scut.edu.cn (J.Z.)

[2] School of Microelectronics, South China University of Technology, Guangzhou 511442, China; 202264680107@mail.scut.edu.cn

* Correspondence: yechen@scut.edu.cn (Y.C.); yunquanli@scut.edu.cn (Y.L.)

† These authors contributed equally to this work.

‡ These authors contributed equally to this work.

The PDF file includes

Section S1. The CAD drawings and parts list of MSESM

Figure S1. CAD model of MSESM

Table S1. Parts list of MSESM

Table S2. The accuracy and workspace comparison of different continuum robots

Table S3. The stiffness performance and rate of change comparison of different continuum robots

Supplementary Materials

Section S1. The CAD drawings and parts list of MSESM

Figure S1 presents a detailed, dimensioned Computer-Aided Design (CAD) model of the Multi-Segment Extendable Soft Manipulator (MSESM) to enhance the reproducibility of the work. The figure illustrates the manipulator's core two-segment structure, composed of the proximal C1 bellows and the distal C2 bellows. Key dimensions are explicitly labeled, including the 80 mm diameter of the end plate, the 50 mm and 60 mm diameters of the respective bellows segments, and the nominal lengths of the C2 (265 mm) and C1 (321 mm) segments. This provides a clear blueprint of the manipulator's geometry and scale.

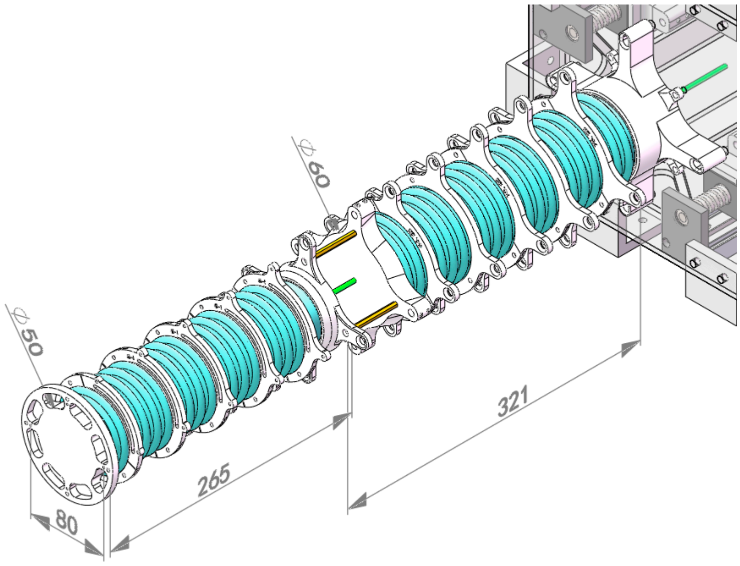

Figure S1. CAD model of MSESM

Table S1 serves as a comprehensive parts list, or Bill of Materials, for the MSESM, detailing the primary components required for its construction. The list includes two custom-sized silicon bellows that form the manipulator's body, four Teflon tubes and four nylon ropes that act as the tendons for the hybrid actuation system, and the primary drive components consisting of four electric sliding rails and four servos. The table also notes the use of 3D-printed components, which can be flexibly adapted based on specific assembly requirements.

Table S1. Parts list of MSESM

Material	Quantity
3D printing component	Flexible replacement according to size adjustment
Silicon bellows	2
Teflon tubes	4
Nylon ropes	4
Electric sliding rails	4
Servos	4

Table S2 provides a comparative analysis of the MSESM's positioning accuracy and workspace against six other state-of-the-art continuum robots from recent literature. The table benchmarks performance using metrics like percentage error for specific tracking tasks and the dimensions of the operational workspace in millimeters. This comparison highlights that while some specialized manipulators achieve higher precision on specific tasks, the MSESM offers one of the largest and most reconfigurable workspaces, with a horizontal extension range of 445-820 mm and a vertical range of 750-1220 mm.

Table S2. The accuracy and workspace comparison of different continuum robots.

Number	Name	Accuracy	Workspace
1	Multi-Segment Extendable Soft Manipulator Driven by a Pneumatic – Tendon Coupling Mechanism (This work)	1.43% (Rect.), 5.19% (Tri.), 24.19% (Circ.)	Horizontal: 445-820 mm Vertical: 750-1220 mm
2	Disturbance-Adaptive Tapered Soft Manipulator with Precise Motion Controller for Enhanced Task Performance with Precise[32]	0.012% (Circular)	Horizontal: 400-600 mm Vertical: 200-800 mm
3	Easy-to-Deploy Combined Nasal/Throat Swab Robot With Sampling Dexterity and Resistance to External Interference[33]	13.8% (Circular)	Horizontal: 300-600 mm Vertical: 250-400 mm
4	Design of a Bio inspired Extensible Continuum Manipulator with Variable Stiffness[7]	Accuracy data missing	Vertical Range: 91-219 mm
5	An Underwater Robotic System With a Soft Continuum Manipulator for Autonomous Aquatic Grasping[4]	0.25%(total)	Length: 265 mm<br>Width: 240 mm<br>Height: 215 mm
6	A 3D printed variable cross-section pneumatic soft manipulator with high-precision positioning capability: Design and control implementation[34]	0.27%(total)	Length & Width: 150 mm<br>Height: 250 mm
7	Design and development of a soft robotic manipulator[35]	Accuracy data missing	Length Range: 274-306 mm

Table S3 offers a quantitative comparison of the stiffness modulation capabilities of the MSESM with other soft manipulators from the literature, showing the range of stiffness performance and the percentage rate of change. The data shows that the MSESM achieves a significant axial stiffness change of 1056% (from 0.22 to 2.5446 N/mm) and a lateral stiffness change of 3834% (from 0.0091 to 0.358 N/mm), demonstrating the effectiveness of its hybrid actuation mechanism for stiffness tuning.

Table S3. The stiffness performance and rate of change comparison of different continuum robots.

Number	Name	Stiffness Performance	Rate of change
1	Multi-Segment Extendable Soft Manipulator Driven by a Pneumatic – Tendon Coupling Mechanism (This work)	Axial:0.22-2.5446N/mm<br>Lateral:0.0091-0.358N/mm<br>Torsional:0.013-0.0145Nm/deg	Axial: 1056%<br>Lateral:3834%<br>Torsional:11.5%
2	Disturbance-Adaptive Tapered Soft Manipulator with Precise Motion Controller for Enhanced Task Performance with Precise[32]	Axial:0.57-10.77N/mm<br>Lateral:0.01-0.45N/mm<br>Torsional:0.02-0.044Nm/deg	Axial: 1889%<br>Lateral:4500%<br>Torsional:120%
3	Design of a Bio inspired Extensible Continuum Manipulator with Variable Stiffness[7]	Lateral(S1):0.011-0.11N/mm<br>Lateral(S2):0.006-0.275N/mm	Lateral(S1):900 %<br>Lateral(S1):4483 %
